# Prevalence of *Dirofilaria immitis* in mosquitoes (Diptera) – systematic review and meta-analysis

**DOI:** 10.21307/jofnem-2021-012

**Published:** 2021-02-19

**Authors:** Seyed Mohammad Riahi, Mustapha Ahmed Yusuf, Shahyad Azari-Hamidian, Rahmat Solgi

**Affiliations:** Cardiovascular Diseases Research Center, Department of Epidemiology and Biostatistics, School of Medicine, Birjand University of Medical Sciences, Birjand, Iran; Department of Medical Microbiology and Parasitology, College of Health Sciences, Bayero University, Kano, Nigeria; Department of Health Education, Research Center of Health and Environment, School of Health, Guilan University of Medical Sciences, Rasht, Iran; Infectious Disease Research Center, Birjand University of Medical Sciences, Birjand, Iran; Cellular and Molecular Center, Birjand University of Medical Sciences, Birjand, Iran

**Keywords:** Meta-analysis, Systematic review, Culicidae, Dirofilariasis, Diagnostic methods

## Abstract

Knowledge of the vectors of dirofilariasis in the world beside the treatment of infected dog is crucial to establish mosquito vector-based control programs. The current systematic review and meta-analysis were conducted on published studies, documenting the prevalence of *Dirofilaria immitis* infected/infective mosquitoes from field surveys and laboratory experiments under controlled conditions. Articles up through 2019 from Scopus, PubMed, Embase, Web of Science, Google Scholar were screened systematically. The overall prevalence of *D. immitis* infected/infective mosquitoes was estimated using a random effect model. Meta-regression was used to identify factors related to high dirofilariasis prevalence in the vectors. In these studies, the detection method was not identified as a heterogeneity and the overall prevalence in both subgroups had overlap (7.9-34.9 and 1.5-48.5). The overall prevalence of infective stage was 2.6 (95% CI: 0.97-4.77 per 1,000) and 84.7 per 1000 (95% CI: 20.5-183.8 per 1,000) for the field survey/laboratory experiment, respectively. The higher overall prevalence of *D. immitis* infected/infective mosquitoes were reported across studies in which take place in Eastern Mediterranean Region office (EMRO), longitude: 80 to 110, latitude: 20 to 40, annual rainfall: 250 to 1000, sea level: 26 to 100 and <1,000, humidity: 66 to 70, during 2000 to 2005 by dissection methods. Our review determined that mosquito species within the genus *Anopheles* and to a less extent *Culex* were the main vectors of dirofilariasis.

Diroﬁlariasis is a zoonotic vector-borne disease transmitted by at least 27 species of the genus *Diroﬁlaria* (Spirurida, Onchocercidae), especially *D. repens* and *D. immitis* (canine or dog heartworm). The disease is widely distributed and the reservoirs are mainly mammals from approximately 111 species including canids. Presently, diroﬁlariasis in humans is considered to be an emerging disease in some regions (Azari-Hamidian et al., 2007, 2009; Simón et al., 2012). At present, at least 77 mosquito species (Diptera: Culicidae) of the genera *Culex*, *Aedes*, *Anopheles*, *Mansonia*, *Coquillettidia*, *Psorophora*, and *Culiseta* are presumed to have a role in the transmission of dirofilariasis, while the third-stage infective larvae (L3) have been detected from in a few field-captured dipteral species (Azari‐Hamidian et al., 2009). The mosquitoes can become infected by ingestion of blood meal from microfilaremic host. The ingested microfilaria can develop to infected (L1, L2) and subsequently into the infective larvae (L3) in the Malpighian tubules. The L3 migrate to the mosquito proboscis. The L3’s transmit through mosquito bite and become sexually mature in the pulmonary artery and right ventricles of suitable mammalian final host (Ledesma and Harrington, 2011). Heartworm disease is endemic disease, mainly locate in regions with temperate and tropical climate. The transmission of dirofilariasis from endemic to the new area directly depend on availability of microfilaremic hosts, competent vectors as well as a favored ecological factors for development of larval stages to L3 in the mosquitoes (Sassnau et al., 2014). The role of ecological key factors has been proposed for development of dirofilarial larvae in possible vectors for specific locations (Brown et al., 2012). Most of the field studies only reported the prevalence in mosquitoes. So, it is important to perform a comprehensive study to evaluate these key factors. The monitoring of mosquitoes for the infection was based on dissection method which is the gold standard (Latrofa et al., 2012). The experimental studies allow the evaluation of vector competence of suspected mosquitoes for transmission of dirofilariasis (Nayar and Knight, 1999). The results of such experimental studies have been recorded, but no comprehensive evaluation of vector efficiency has been done in all mosquitoes to date. The current systematic review and meta-analysis were conducted on published studies, documenting the prevalence of *D. immitis* infected (L1-L2)/infective(L3) mosquitoes from field surveys, examine the effect of different key factors on their overall prevalence and comprehensively assess the vector competence from experimental studies.

## Material and methods

Research was conducted in accordance with Systematic Reviews and Meta-Analysis (PRISMA) 2015 Guidelines (Moher et al., 2015).

### Search strategy and study design

Original search studies that evaluated the mosquito vectors for *D. immitis* in field and experiment were screened in PubMed, Embase, Web of Sciences, Scopus, and Google Scholar up to October 10, 2019. The keys terms used for searching in literature were ‘*Dirofilaria immitis’*, ‘vector’, ‘prevalence’, ‘Culicidae’, and ‘mosquitoes’ with the Boolean operators ‘OR’ and/or ‘AND’. The manually searched in the reference lists of the collected studies was also performed. After removal of duplicate articles, the retrieved papers were screened by title and abstract by two independent authors to exclude any that were irrelevant to our study question (S. R. and R. SM). The full text of the remaining articles was evaluated to identify eligible articles. Studies were considered eligible if they had cross-sectional design. Results were screened initially excluding all studies that did not included *D. immitis* and vectors. The other exclusion criteria were: non original paper (review, systematic reviews, case reports, letter or editorial articles, thesis), unidentified filarial species, undefined data, and lacking control groups for experimental studies. The experimental vector competence studies as well as the field studies were included.

### Data extraction

Data were extracted from published articles, evaluating the prevalence of *D. immitis* infected/infective mosquitoes from field and experimental studies in an Excel form (Microsoft, Redmond, WA, USA). The data extraction was performed by S. R. and R. SM. and in cases of discrepancy, consensus was carried out by discussion with A. S. Subsequently, an author (S. R) extracted the requisite data, and the others (R. SM. and A. S.) rechecked them. Data analysis was performed by R. SM. For field study, the following items were extract: ‘General information’ including first author, year of implementation (The years for collection of samples), and location; geographical data of sampling site including average temperature, annual rainfall, humidity, WHO regional office, altitude, latitude, longitude; ‘vectorial characteristics’ including number of samples, total number of pools, total number of *D. immitis* infected/infective mosquitoes. In some studies, mosquitoes were evaluated individually for both head+thorax and abdomen in order to differentiate infective and infected specimens, and in others, different parts of mosquitoes were studied in pools. In order to consistency of calculations, in studies in which mosquitoes were studied individually each mosquito was considered as a one pool. So, pool in this study defines as a number of one or more mosquitoes that were placed in a one cluster and considered as a unit of study. The total number of infective and infected pool of mosquitoes was calculated as a number of pools that have an infective and infected larvae stages, respectively. Minimal infected/infective rate, calculated as the ratio of the number of positive pools of infected/infective mosquitoes to the total pool of tested mosquitoes, assumes that there is only one infected/infective individual in each positive pool. Since the nominator and denominator of fraction were the same, it has little effect on the infected/infective rate. For experiment study, the general information and vectorial characteristics were extracted. To quantitatively evaluate vector competence, overall prevalence of *D. immitis* infection and infective stage in filed and experiment collected mosquitoes was performed.

### Quality assessment

Evaluation of the included study quality was conducted by two authors (R. SM and S. R.) independently using the Newcastle-Ottawa Scale (Stang, 2010). Brieﬂy, maximum of nine scores was deﬁned for the following items including the subject selection criteria (0-4 points), comparability of subjects (0-2 points), and exposure (0-3 points). The papers with a total score of 0 to 3, 4 to 6, and 7 to 9 points were speciﬁed as the poor, moderate, and high quality, respectively.

### Data synthesis and statistical analysis

The STATA software version 13 was used for meta-analysis (Stata Corp LP, College Station, Texas). The main goals were the evaluation of prevalence of *D. immitis* in field collected vectors and assessment the competence of suspected vector for *D. immitis* in experimental studies by dissection and molecular methods. The evaluation of field and experimental studies was performed separately. First, random effects model was used for calculation of overall estimates. Metaprop was used for calculating overall prevalence using a Freeman–Tukey double arcsine transformation and then confidence intervals (CI = 95%) for each survey were assessed (Freeman and Tukey, 1950; Harris et al., 2008; Nyaga et al., 2014). Next, the key factors that could be the source of heterogeneity for the prevalence of infection and infective were assessed using meta-regression and subgroup analyses for some factors such as: data collection years, country, mean temperature, annual rainfall, humidity, WHO regional office, altitude, latitude, detection type, vector genus, and vector species. Heterogeneity among studies was evaluated by statistical tests and graphical (i.e., Cochran’s *Q* test, *I*^2^ statistics, and Galbraith). The range of *I*^2^ was between 0 and 100%. *I*^2^ > 70% were considered heterogeneous (Higgins, 2008; Riahi and Mokhayeri, 2017). In this study the relationship between exposures and outcome was not studied, so evaluation of publication bias was not logical (Mokhayeri et al., 2018). The significance level of ≤0.05 was considered.

## Results

### Inclusion of studies and data extraction

**Figure 1:**
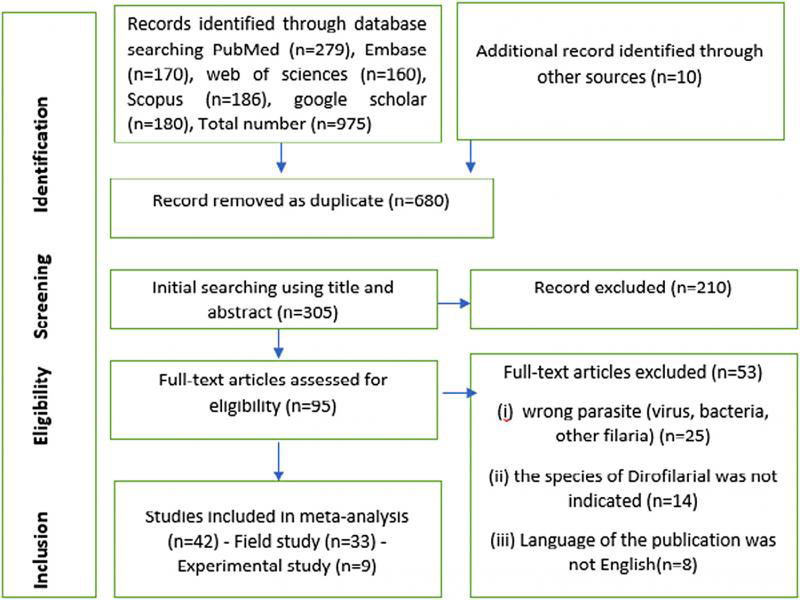
Flow chart detailing the number of studies excluded and included at each step for systematic review of the prevalence of *D. immitis* infection in Culicidae mosquitoes.

A flow chart for identification, screening, exclusion, and finally including of retrieved article was shown in Figure 1. A total of 985 literatures was at first screened that of which 680 were removed as duplicated. After that, initial searching using title and abstract (*n* = 305 articles) was performed. After screening, 210 articles were excluded and full text of remaining papers (*n* = 95) reviewed for evaluating of eligibility, which of those 53 record removed (Fig. 1). From 42 included articles, 33 and 9 studies were field studies and laboratory experiments, respectively. The selected articles from field study involved 19 countries from Pan American Health Organization (PAHO) (21%), European Region (EURO) (52.6%), Eastern Mediterranean Regional Office (EMRO) (5.2%), and Western Pacific Regional Office (WPRO) (21%). Studies were published over a 29-year period (1990-2019). Molecular assay was used in 9 out of 33 field study and 3 out of 9 experimental studies to identify the *D. immitis* larvae in vectors (Tables 1, 2).

**Table 1.** Characteristics of the individual studies included in the meta-analysis based on eligibility criteria.

Country	Year of implementation	Method	Total sample	Total pool	Total infected pool	Total infective pool	Quality score	References
Brazil	2013	PCR	3115	311	1	0	7	Ogawa et al. (2013)
Mexico	2015	PCR	2618	595	4	0	6	Oswaldo et al. (2018)
USA	2006	PCR	1,922	390	33	0	7	Chambers et al. (2009)
Mexico	2007	Dissection	272	272	28	21	8	Manrique-Saide et al. (2008)
Italy	2012	PCR	40,892	955	21	0	7	Latrofa et al. (2012)
Iran	2005-2011	PCR	190	190	15	10	6	Azari-Hamidian et al. (2009)
USA	2009	PCR	91,798	1,212	184	54	8	Mckay et al. (2013)
Germany	2011-2013	PCR	16,878	955	2	0	7	Kronefeld et al. (2014)
Italy	2000-2002	PCR	3,198	1,364	10	4	6	Cancrini et al. (2006)
Brazil	1995-1996	Dissection	3,677	3,677	55	8	7	Labarthe et al. (1998)
Portugal	2011-2013	PCR	5,866	1,815	54	23	8	Ferreira et al. (2015)
Argentina	2003-2005	Dissection	2,380	2,380	3	0	7	Vezzani et al. (2006)
Russia	2007-2017	PCR	387	78	5	0	8	Shaikevich et al. (2018)
USA	1994	Dissection	188	188	3	0	6	Comiskey and Wesson (1995)
Italy	2002-2003	PCR	1,576	880	6	3	5	Cancrini et al. (2007)
Italy	2003	PCR	154	154	2	1	6	Cancrini et al. (2003a, 2003b)
Italy	2000-2002	Dissection	2,534	336	81	22	5	Cancrini et al. (2003a, 2003b)
Spain	2005-2007	PCR	1,219	1,219	1	0	7	Morchón et al. (2011)
Argentina	2007-2008	PCR	453	82	2	0	6	Vezzani et al. (2011)
Portugal	2002-2003	PCR	554	554	4	2	7	Santa-Ana et al. (2006)
Turkey	2008-2009	PCR	6,153	559	15	14	8	Yildirim et al. (2011)
Spain	2012-2013	PCR	881	881	1	1	6	Bravo-Barriga et al. (2016)
Turkey	2008	PCR	301	54	2	1	8	Bıs¸kın et al. (2010)
Slovakia	2013	PCR	10,500	105	4	0	8	Bocková et al. (2015)
Spain	2004-2006	PCR	725	725	2	0	7	Morchon et al. (2007)
USA	1985-1987	Dissection	9,377	9,377	123	18	7	Parker (1993)
South-Korea	2005	PCR	2,059	172	12	0	6	Lee et al. (2007)
USA	2006-2007	PCR	1,401	206	15	0	8	Licitra et al. (2010)
France	2015	Real time PCR	797	797	29	0	8	Tahir et al. (2017)
Italy	2005	PCR	637	119	1	0	7	Masetti et al. (2008)
Taiwan	1997	Dissection	4,537	4,537	146	0	6	Lai et al. (2001)
Samoa	1978-1980	Dissection	41,980	41,980	179	49	7	Samarawickrema et al. (1992)
French Polynesia	2003-2004	Dissection	1,194	1,194	28	4	8	Russell et al. (2005)

**Table 2.** List of published articles included in the systematic review for experiment studies of *D. immitis* in vectors.

Country	Year of implementation	Detection method	Total sample	Total pool	Total infected pool	Total infective pool	Quality score	References
Brazil	2000	Dissection	756	756	225	57	8	Ahid et al. (2000)
Italy	2017	PCR	136	136	66	10	7	Silaghi et al. (2017)
Italy	2015	PCR	45	45	37	18	8	Montarsi et al. (2015)
Iran	2017	PCR	90	90	58	16	7	Solgi et al. (2017)
Brazil	2008	Dissection	1,942	1,942	465	0	8	Carvalho et al. (2008)
USA	2005	Dissection	750	750	630	0	8	Debboun et al. (2005)
Brazil	1998	Dissection	73	73	43	0	7	Macêdo et al. (1998)
Australia	1989	Dissection	215	215	0	49	6	Russell et al. (2005)
Brazil	1999	Dissection	505	505	0	101	8	Brito et al. (1999)

### Meta-analysis for estimating overall prevalence of *Dirofilaria immitis* infected/infective stages in the filed collected mosquitoes

Meta-analysis was performed for estimating overall prevalence of *D. immitis* infected/infective stages in the genera and species of field collected mosquitoes (Table 3). The magnitude of the overall prevalence differed largely across all mosquito genera and species. When reporting overall prevalence of *D. immitis* infected stages in vectors, it ranged between 6.7 per 1000 (95% CI: 3.48-10.88 per 1000) in *Cx. pipiens* complex and 49.5 per 1,000 (95% CI: 14.7-101.6 per 1000) in *Ae. albopictus*. The overall prevalence of *D. immitis* infected stages across all mosquito species was 21.8 per 1000 (95% CI: 14.06-31.18 per 1,000) and heterogeneity was substantial (*I*^2^ = 98.5%). Subgroup analysis for infective rate in vector produced overall prevalence ranging from 0 per 1,000 in *Ochlerotatus sollicitans* to 19.2 per 1,000 in *Cx. theileri* (95% CI: 0.06-62.5 per 1,000). The overall prevalence of *D. immitis* infective stages across all mosquito species was 2.6 per 1,000 (95% CI: 0.97-4.77 per 1,000) and heterogeneity was substantial (*I*^2^ = 94.4%).

**Table 3.** Subgroup meta-analysis of studies reporting *Dirofilaria immitis* infected and infective rates in vectors grouped by mosquito genus and species.

		Minimum infected rate^a^				Minimum infective rate^b^			
Variable	pool size	Positive pool	Overall prevalence (95% CI)	*I*^2c^	*Q*	Positive pool	Overall prevalence (95% CI)	*I*^2^	*Q*
Total	124,480	1,071	21.86 (14.06-31.1)	98.5	2,475.9	235	2.6 (0.97-4.77)	94.4	622.1
*Aedes*	62,924	526	29.5 (17.21-46.4)	97.5	1,016.7	138	1.8 (0.03-5.3)	91.6	298.4
*Culex*	16,652	260	12.36 (5.8-20.88)	92.9	241.8	42	1.28 (0.0-4.04)	83.3	95.6
*Anopheles*	4,244	157	51.37 (5-133.5)	98.1	372.9	19	3.14 (0.0-12.2)	75.5	28.6
*Ochlerotatus*	5,382	73	43.29 (0.1-140.6)	96.3	54.9	24	0.97 (0.14-2.27)	0.0	–
*Cx. quinciafasiatus*	4,892	168	24.7 (7.79-49.4)	92	62.7	13	2.6 (0.0-12.46)	87.6	40.2
*Ae. polynesiensis*	24,041	198	31.8 (3.5-85.5)	98	100.8	53	1.8 (0.5-3.86)	32.7	2.9
*Oc. taeniorhynchus*	2,517	44	12.6 (8.42-17.5)	0.0	–	32	4.1 (1.73-7.31)	0.0	–
*Oc. sollicitans*	2,539	25	0	–	–	2	0	–	–
*Cx. pipiens*	5,518	43	6.7 (3.48-10.88)	59.7	19.8	7	0.62 (0-2.29)	48.3	15.5
*Ae. ochcaspius*	285	14	48.6 (25.81-77.6)	0.0	–	4	10.7 (0.91-27.7)	0.0	–
*Ae. vexans*	13,439	77	47.1 (1.08-135)	97.6	299.3	30	6.2 (0.0-38.53)	94.0	117.2
*Cx. theileri*	1,574	43	35.3 (2.06-101.4)	96.2	78.5	25	19.2 (0.06-62.5)	94.5	54.3
*Ae. albopictus*	2,298	151	49.5 (14.7-101.6)	95.3	169.7	25	3.2 (0.00-15.30)	86.3	58.6

**Notes:** ^a,b^The result present as a case per 1,000; ^c^heterogenicity index.

### Meta-analysis for evaluating risk factor on overall prevalence of *Dirofilaria immitis* infected/infective stages in vectors

Different factors were analyzed on overall prevalence of *D. immitis* infected/infective stages in vectors (Tables 4, 5). The overall prevalence of dirofilariasis decreased with implementation years from 2000 to 2019. Dirofilariasis prevalence differs between countries. The overall prevalence of *D. immitis* infective stages in vectors ranged from 1.4 per 1,000 (95% CI: 0.3-3 per 1,000) in Brazil to 52.6 per 1,000 (95% CI: 25.5-94.7 per 1,000) in Iran. The greatest overall prevalence of infective rate was reported across studies conducted in the Eastern Mediterranean Region, longitude 80 to 110 (12.5 per 1,000, 95% CI: 0.0-49 per 1,000), latitude 20 to 40 (5.7 per 1,000, 95% CI: 1.3-12.2 per 1,000), annual rainfall 250 to 500 millimeter (5.1 per 1,000, 95% CI: 1.4-10.2 per 1,000), sea level 26 to 100 (11.2 per 1,000, 95% CI: 0.3-34.6 per 1,000) and <1,000 meter (10 per 1,000, 95% CI: 0.0-35.1 per 1,000), humidity 66-70% (7.4 per 1,000, 95% CI: 1.2-18 per 1,000) during 2000 to 2005 (9.6 per 1,000, 95% CI: 0.0-36.4 per 1,000) by dissection methods (4.5 per 1,000, 95% CI: 1.6-8.6 per 1,000). The mean temperatures of collection sites vary from 7 to 28°C. The higher infective rate was report between 15 and 21°C.

**Table 4.** Subgroup meta-analysis of field studies reporting pool prevalence *Dirofilaria immitis* infected/infective rates in vectors grouped by country.

		Minimum infected rate^a^			Minimum infective rate^b^		
Variable	Pool size	Positive pool	Overall prevalence (95% CI)	*I*^2c^	Positive pool	Overall prevalence (95% CI)	*I*^2^
Global	124,480	1,071	21.9 (14-31.2)	98.6	235	2.6 (1-4.8)	94.4
*PAHO*	18,690	451	32.4 (13.3-59)	98.2	101	3.8 (0.1-11.1)	95.3
Argentina	2,462	5	0 (0-1.4)	99.9	0	0 (0-0.3)	99.8
Brazil	3,988	56	13.2 (9.8-17.1)	99.9	8	1.4 (0.3-3)	99.8
Mexico	867	32	25 (15.4-36.8)	99.9	21	9.4 (3.7-17.3)	99.8
USA	11,373	358	57.9 (8.1-146.5)	99	72	4 (0-23.4)	96.8
*EURO*	57,717	240	14.8 (5-28.7)	97.2	71	2.2 (0.1-6.2)	92.9
Italy	3,808	121	30.3 (3.1-80.6)	97.7	30	6.3 (0-20.8)	91.9
Portugal	2,389	58	23.1 (17.4-29.6)	99.5	25	10 (6.3-14.5)	98.4
Slovakia	187	4	14.7 (0.9-39.4)	99.5	0	0 (0-10.2)	98.4
Spain	2,825	4	1.3 (0.2-3.2)	0	1	0.1 (0-1.3)	0
Turkey	613	17	24.5 (12.7-39.3)	5.1	15	21.4 (10.4-35.5)	34.2
Czech	237	0	0 (0-15.4)	0	0	0 (0-15.4)	0
France	797	29	36.4 (24.5-51.8)	–	0	0 (0-4.6)	–
Germany	955	2	2.1 (0.3-7.5)	–	0	0 (0-3.9)	–
Russia	78	5	64.1 (21.2-143)	–	0	0 (0-46.2)	–
Australia	2,389	0	0 (0-0.1)	–	0	0 (0-0.1)	–
*EMRO*	190	15	78.9 (44.9-126.9)	–	10	52.6 (25.5-94.7)	–
Iran	190	15	78.9 (44.9-126.9)	–	10	52.6 (25.5-94.7)	–
WPRO	47,883	365	25.6 (6.1-57.4)	98.9	53	0.3 (0-2.0)	81.2
French Polynesia	1,194	28	23.5 (15.6-33.7)	–	4	3.4 (0.9-8.6)	–
Samoa	41,980	179	4.3 (3.7-4.9)	–	49	1.2 (0.9-1.5)	–
Taiwan	4,537	146	32.2 (27.2-37.7)	–	0	0 (0.0-0.8)	–
South-Korea	172	12	69.8 (36.6-118.7)	–	0	0 (0.0-21.2)	–

**Notes:** ^a,b^The result presents as a case per 1,000; ^c^heterogenicity index.

**Table 5.** Subgroup meta-analysis of field studies reporting pool prevalence *D. immitis* infected and infective rates in vectors grouped by risk factor.

		Minimum infected rate^a^			Minimum infective rate^b^		
Variable	Pool size	Positive pool	Overall prevalence (95% CI)	*I*^2c^	Positive pool	Overall prevalence (95% CI)	*I*^2^
*Latitude*							
0-20	44,470	245	16.9 (3.2-40.1)	96.6	53	0.5 (0.2-0.9)	1.8
20-40	25,560	609	29 (15.1-46.9)	97.7	129	5.7 (1.3-12.2)	95.1
>40	54,450	217	17.4 (4.5-37)	98.3	53	1.70 (0-6.1)	93.1
*Longitude*							
0-10	4,218	86	13.1 (1.3-36)	95.7	24	1.9 (0-9.5)	90.9
10-20	52,621	128	12 (1.8-29.6)	98.1	32	2.1 (0-6.7)	93.3
20-50	4,745	96	29.9 (12.9-52.6)	81.9	33	8.4 (0-26-9)	87.8
50-80	12,150	129	6.1 (0-18.8)	93.7	18	0 (0-1.4)	66.9
80-110	2,473	234	58.1 (9.1-143)	97.7	75	12.5 (0-49)	96
>110	48,273	398	34.8 (11.5-69.4)	98.8	53	0.3 (0-1.7)	75.1
*Mean temperature °C*							
7-14.9	52,431	190	23.3 (7.3-46.9)	98	47	2.5 (0-8.1)	92.3
15-21.9	18,905	404	19.6 (6-39.9)	98.1	106	4.1 (0.4-10.4)	94.2
22-28.1	53,144	477	23.9 (10.8-41.5)	98.1	82	1.9 (0.1-5.1)	90.9
*Annual rainfall (mm)*							
<250	49,029	5	0.4 (0-3)	79.5	1	0 (0-0.1)	37.3
251-500	24,280	509	27.8 (16.6-41.5)	96	100	5.1 (1.4-10.2)	92
501-1,000	8,801	345	32 (9.8-65.7)	97.9	85	3.4 (0-12.7)	94.9
>1,000	42,370	212	3.9 (3.3-4.6)	99.7	49	0.6 (0.3-0.9)	99.3
*Sea level (m)*							
<25	15,367	281	28.7 (12.4-50.9)	96.4	52	3.4 (0.2-9.1)	87.8
26-100	6,945	291	36.1 (4.7-93.7)	98.9	98	11.2 (0.3-34.6)	97.6
101-200	95,282	379	13.3 (4.1-26.8)	99.2	57.9	0 (0-1.1)	93.7
201-1000	4,067	58	12.3 (1.4-31.7)	94	3	0.1 (0-1)	0
>1,000	2,819	62	27.8 (4.4-67.3)	95.1	25	10 (0-35.1)	93.8
*Mean humidity (%)*							
<65	5,171	73	12.3 (1.5-29.6)	89.5	24	5.8 (0-19.6)	89.6
66-70	15,821	413	26.9 (6.1-61.1)	98.8	104	7.4 (1.2-18)	95.9
71-75	51,514	132	24.4 (8.2-48.1)	97.6	31	1.8 (0-7.1)	91
76-80	8,579	235	22.2 (9.9-38.8)	93.1	27	1.2 (0-8.5)	93.7
>80	43,395	218	14.1 (0.9-39.4)	95.3	0.49	0.1 (0-0.3)	0
*Detection method*							
PCR	60,539	425	19.3 (7.9-34.9)	98.3	113	2 (0-5.7)	93.7
Dissection	63,941	646	30.5 (1.5-48.5)	98.8	122	4.5 (1.6-8.6)	95.2
*Implementation year*							
1992-2000	55,222	360	10.5 (4-19.7)	97.3	75	0.9 (0.4-1.6)	40.7
2000-2005	6,221	257	57.3 (14.5-124.7)	98	27	9.6 (0-36.4)	96.6
2006-2010	8,525	132	24.4 (10.1-43.9)	95.3	41	3.7 (0.1-10.3)	89.6
2011-2015	6,313	283	19.9 (2.6-50.2)	94.4	91	3.8 (0-14.6)	93.5
2016-2018	48,199	39	10.3 (0–35.1)	97.4	1	0 (0-0.1)	45.4

**Notes:** ^a,b^The result presents as a case per 1,000; ^c^heterogenicity index.

### Meta-analysis of experimental studies for overall prevalence of *Dirofilaria immitis* infected/infective stages in mosquitoes

The total sample size of experimental studies was 4,512. The overall prevalence of *D. immitis* infected and infective stages in mosquitoes were 397 per 1,000 (95% CI: 154.5-636.5 per 1,000) and 84.7 per 1,000 (95% CI: 20.5-183.8 per 1,000), respectively. The PCR technique was more sensitive than morphological tests. *Anopheles* spp were accused as a more competent vector than other mosquitoes for transmission of *D. immitis* (Table 6).

**Table 6.** Subgroup meta-analysis of experimental studies reporting *Dirofilaria immitis* infected and infective rates in vectors grouped by mosquito genus and species.

		Minimum infected rate^a^			Minimum infective rate^b^		
Variable	Pool size	Positive pool	Overall prevalence (95% CI)	*I*^2^	Positive pool	Overall prevalence (95% CI)	*I*^2^
Overall	4,512	1,524	379.9 (154.5-636.5)	89.2	251	84.7 (20.5-183.8)	98.8
Method PCR	271	161	649.2 (459.5-817.8)	99.7	44	194.6 (55-388.8)	99.4
Method Dissection	4,241	1,363	253.7 (40.4-565.7)	99.6	207	46.9 (0.9-147)	99.1
*Ae.* spp.	1,506	905	482.2 (105.2-871.6)	99.6	168	132.4 (17.3-324.2)	98.5
*Cx.* spp.	2,893	561	61.2 (0.5-197)	98.8	65	55.9 (2.7-163.1)	98.2
*An.* spp.	90	58	644.4 (536.5-742.6)	–	16	177.8 (105.2-274.6)	–
*Oc.* spp.	0	0	0		0	0	

**Notes:** ^a,b^The result presents as a case per 1,000.

## Discussion

### Summary of evidence

This is the first study that aimed to summarizing information from field and experiments studies related to overall prevalence of *D. immitis* infected/infective mosquitoes using a meta-analysis. The potency of mosquito species to become infected and then transmit *D. immitis* is an important step that led to the accurate measurement the role of vectors in transmission (Morchón et al., 2012). Based on our result, the prevalence of infective stages of *D. immitis* in field mosquitoes was different according to genus and species. The most infective rate belongs to *Anopheles* spp., *Aedes* spp., and *Culex* spp. with the overall prevalence of 3.1 per 1,000 (95% CI: 0-12.2 per 1,000), 1.8 per 1,000 (95% CI: 0.03-5.3 per 1,000), and 1.2 per 1,000 (95% CI: 0-4.4 per 1,000), respectively. Due to the higher species diversity of *Anopheles* compare to other studied genera and the high infective rate among species of this genus, it was expected to have the highest rate of infectivity for *Anopheles* spp. Interestingly, the overall prevalence of the *D. immitis* infective mosquitoes in experimental studies was the same of field cached mosquitoes in accordance to the genus. *D. immitis* is sub-periodic, with peak density of microfilaria in a host’s peripheral circulation during the evening, presumably synchronizing with circadian cycles of their potential vectors. So, *Culex theileri* with feeding behavior of nocturnal is also among the species with the highest overall prevalence of *D. immitis* infected and infective stages. (Ledesma and Harrington, 2011). The prevalence rate of mosquitoes in field studies were varied in different geographical property, including latitude, longitude, mean temperature, annual rainfall, sea level, and humidity. The factor ‘country’ should be explicate with the many factors such as different national vector control measures and ecological context (Genchi et al., 2009). Diverse ecological and geographical characteristics can also contribute toward biological variation. Climate change together with a shifting weather patterns facilitate the development of mosquitoes vectors of *D. immitis* and faster larvae development on them (Morchón et al., 2012). Today, it was completely determined that climate pattern can affect vector-borne disease transmission. In this meta-analysis study, it was shown that the high infected and infective rate was registered at the temperature optima of 15 to 21°C that it may be due to a suitable temperature for the development of the infectious stages of *D. immitis* in the mosquito. In this regard, Iran with average temperature of 17.5 had a highest registered *D. immitis* infective rate among the world. The extrinsic development is possible above 14°C and it takes e.g. 10 to 12 days at 24 to 26°C (Silaghi et al., 2017). One of the main reasons for decrease of infection rate at higher temperatures despite the appropriateness of the extrinsic period may be due to the high mortality rate of insects (Bayoh and Lindsay, 2004). The other important factors that could influence vector behavior and survival is relative humidity (Brown et al., 2012). Based on the current meta-analysis study, the mean humidity for highest infective rate was 66 to 70%. In this regard, the highest infective rate was belonged to the studies in Iran and Turkey with relative humidity of 72 and 62% at sampling sites. The standard laboratory conditions for transformation of microfilaria to infective larvae on mosquitoes are at a temperature of 25°C and relative humidity of 85% (Silaghi et al., 2017), while it was 15 to 21°C and relative humidity of 66 to 70% for field condition. In the experimental studies, the optimum condition for infection of mosquitoes is provided, so as expected, the overall prevalence of *D. immitis* infection of mosquitoes in the experimental studies is higher than the field studies.

### Strength

To our knowledge, our work is the only study to evaluate an overall prevalence of *D. immitis* in vectors. The other strength of this study were comprehensive literature searches, rigorous methodology, studies from different part of the world, deﬁned clear inclusion and exclusion criteria.

### Limitation

This study has some limitations that are based on the nature of the published studies such as different diagnostic test in included studies. Further, some studies published in local journals may be missed.

## Conclusion

Based on ﬁnding of this study, among different genera of mosquitoes, the genus of *Anopheles* were introduced as a potential vectors of Heartworm disease with the highest infective rate. It must be considered that in addition to the infection rate other factor such as host preference; vector feeding behavior; mortality rates of infected vector; vector and host abundance and microfilaremic reservoir and susceptible host encounter rate are important for affecting transmission of mosquito species (Ledesma and Harrington, 2011). This study showed that the WHO region Number, humidity, longitude, latitude, annual rainfall, sea level, the year of implementation and identification methods may affect the overall prevalence of infected and infective rate.
